#  A 95-year-old patient with unexpected coronavirus disease 2019 masked by aspiration pneumonia: a case report

**DOI:** 10.1186/s13256-020-02432-7

**Published:** 2020-06-23

**Authors:** Francesco Spannella, Letizia Ristori, Federico Giulietti, Serena Re, Paola Schiavi, Piero Giordano, Riccardo Sarzani

**Affiliations:** 1 Internal Medicine and Geriatrics, “Hypertension Excellence Centre” of the European Society of Hypertension, Italian National Research Centre on Aging, Hospital “U. Sestilli”, IRCCS INRCA, via della Montagnola n. 81, 60127 Ancona, Italy; 2 Department of Clinical and Molecular Sciences, University “Politecnica delle Marche”, Ancona, Italy

**Keywords:** SARS-CoV-2, COVID-19, Older, Elderly, Pneumonia

## Abstract

**Background:**

Severe acute respiratory syndrome coronavirus-2 infection has become a pandemic disease (coronavirus disease 2019). The infection has moved from China to the rest of the world and Italy represents one of the most affected countries. Older adults are more susceptible to develop complications with the consequent highest mortality rates.

**Case presentation:**

We report a case of a 95-year-old Caucasian woman affected by pneumonia, initially defined as common aspiration pneumonia in a bedridden patient with vascular dementia, which later turned out to be coronavirus disease 2019 pneumonia during the initial spread of severe acute respiratory syndrome coronavirus-2 in our district. Some features of a computed tomography scan of her chest and her clinical history with known dysphagia had led at first to a different diagnosis with a consequent exposure of health professionals to infectious risk in two distinct hospitals. In this case report, we describe the clinical/imaging features of coronavirus disease 2019 pneumonia and the diagnostic process that led to a correct diagnosis in a nonagenarian with multiple comorbidities.

**Conclusions:**

This case report highlights both the possible pitfalls in diagnosing coronavirus disease 2019 pneumonia in very old patients with comorbidities and the greater than expected spread of the infection, even in individuals with reduced interpersonal contacts and no defined epidemiological link.

## Background

Coronavirus disease 2019 (COVID-19), caused by severe acute respiratory syndrome coronavirus-2 (SARS-CoV-2) infection, has spread globally since the first cases were described in November 2019 in Wuhan, China. Since 21 February 2020, when COVID-19 emerged in northern Italy’s Lombardy region, the number of infected patients in Italy closely followed an exponential trend, with the risk of reaching saturation point for beds in intensive care units. The higher risk of complications and death in older patients was immediately evident [1]. In a Chinese report, older patients accounted for 15.1% of the patients affected by COVID-19 and 27% of patients with severe disease were older than 65 years [2]. The Italian National Institute of Health (Istituto Superiore di Sanità; ISS) found that 38.4% of the patients affected by COVID-19 in Italy were older than 70 years of age. Moreover, the case fatality rate increases with increasing age, reaching 32.7% for those over 80 years of age [3]. Lung injury plays a central role in the pathophysiology and symptoms of COVID-19 [4].

Respiratory diseases and pneumonia per se are among the major causes of hospitalization in the older population with a marked higher mortality rate compared to the adult population [5]. Aspiration pneumonia is a leading cause of pneumonia in older patients, accounting for 5 to 15% of all cases of community-acquired pneumonia [6], and it is estimated that at least 70% of hospitalized pneumonia in patients older than 70 years can be diagnosed as aspiration pneumonia [7].

In this case report, we describe the clinical/imaging features of a case of COVID-19 pneumonia, initially defined as common aspiration pneumonia, and its diagnostic process in a very old patient with comorbidities, when the SARS-CoV-2 infection was not yet widespread in Italy.

## Case presentation

A 95-year-old Caucasian woman was admitted to our Internal Medicine and Geriatrics Department on 2 March 2020. Her symptoms started on 26 February 2020 with onset of fever, cough, and vomiting, which led to her hospitalization in the main general hospital of the town in which she lived, where an initial diagnosis of aspiration pneumonia was made. In the diagnostic workup, she had several comorbidities including arterial hypertension, chronic heart failure, paroxysmal atrial fibrillation, dyslipidemia, stage G4 chronic kidney disease, vascular dementia with deconditioning syndrome, sacral pressure ulcers, and known dysphagia. Home medications were the following: furosemide 25 mg, amiodarone 200 mg, warfarin 5 mg, and pantoprazole 20 mg.

**Fig. 1:**
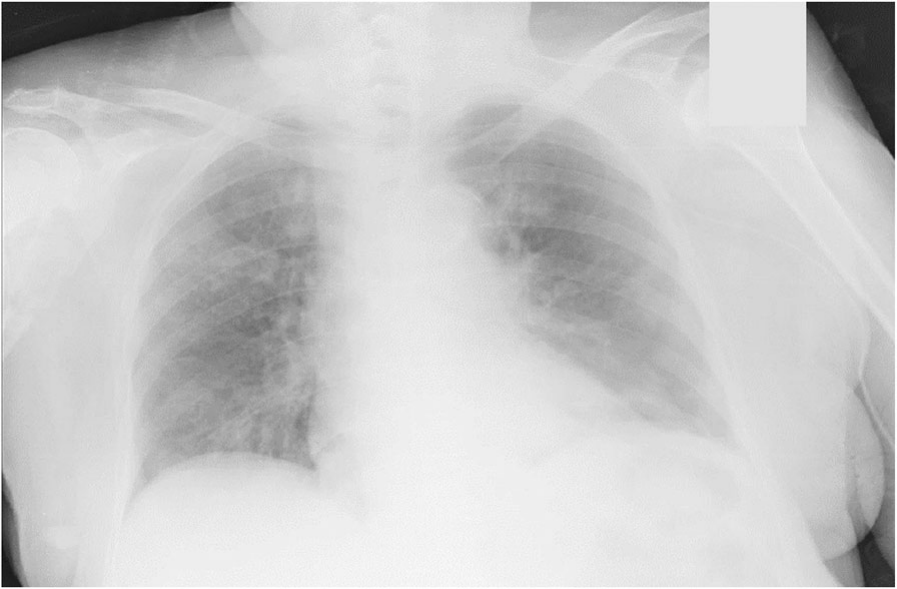
Chest X-ray performed on admission (single supine anteroposterior view) showing bilateral patchy shadowing

**Fig. 2:**
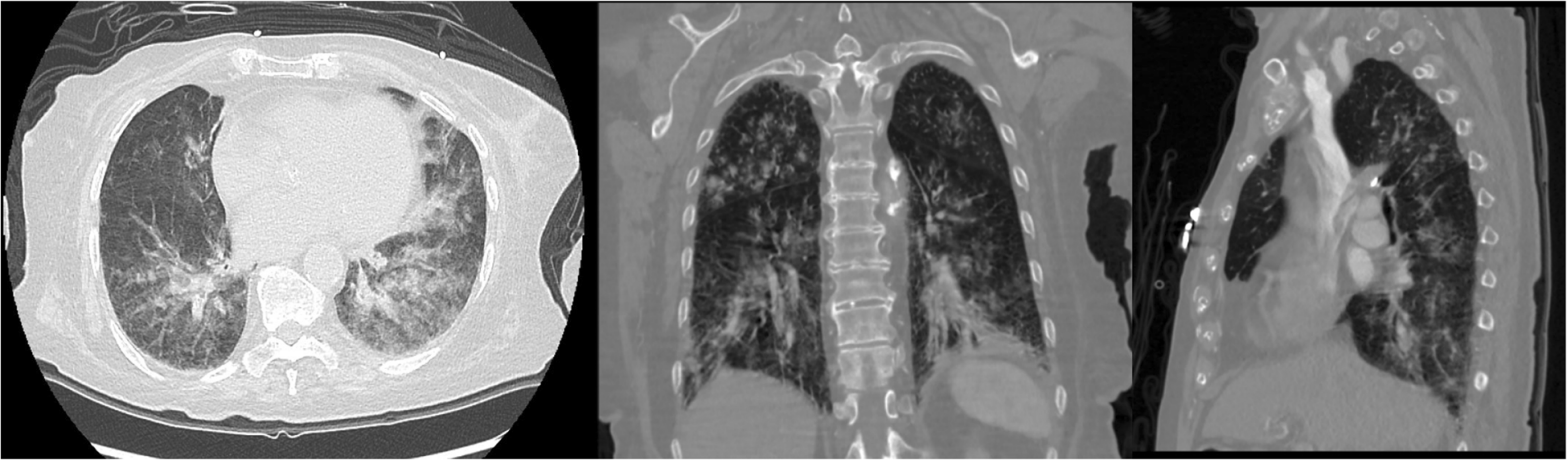
Chest computed tomography performed on day 1 after admission showing multiple bilateral ground glass opacities, coupled with crazy-paving pattern, as well as bilateral lobular and sub-segmental areas of consolidation. Axial view (*left*); coronal view (*center*); sagittal view (*right*)

She lived at home with her son and a caregiver. At initial interview, both of them denied any travel to areas of high transmission for COVID-19 or contact with people coming from these areas (there was no clear epidemiological link). She had been vaccinated for the seasonal influenza virus. She was almost totally dependent on both basic activities of daily living (BADL) and instrumental activities of daily living (IADL). On admission, she had fever (39.1 °C) and acute respiratory failure requiring oxygen supplementation with arterial oxygen saturation (SaO_2_) of 93% with fraction of inspired oxygen (FiO_2_) of 40%, altered mental status, tachycardia with heart rate of 94 beats per minute (bpm), and high blood pressure (160/80 mmHg). Other relevant features on physical examination were bilateral lung crackles and peripheral pitting edema. On day 1 after admission, a chest computed tomography (CT) scan was performed to better characterize the admission chest X-ray findings (bilateral patchy shadowing, Fig. 1), showing multiple bilateral ground glass opacities (GGOs), crazy-paving pattern, and bilateral lobular and sub-segmental areas of consolidation (mainly focused in lingular segment of left lung and inferior lobe of bilateral lungs) (Fig. 2). Laboratory tests on admission (Table 1) showed a normal white blood cell (WBC) count with lymphopenia, high C-reactive protein, and slightly increased serum levels of pro-calcitonin. D-dimer and aminotransferase levels were within normal range, whereas N-terminal pro-B natriuretic peptide (NT-proBNP) levels were elevated. Pneumococcal and *Legionella* urinary antigen tests were negative. Blood and urine cultures were negative. Bronchoalveolar lavage collected from our patient on admission tested positive for methicillin-sensitive *Staphylococcus aureus* (10,000 CFU/ml) and *Citrobacter werkmanii* (100,000 CFU/ml), both sensitive to piperacillin-tazobactam. Based on this information, the empiric antimicrobial therapy started on admission with piperacillin-tazobactam was maintained. A bedside swallowing assessment was also performed, confirming dysphagia to both liquids and solids. During the hospitalization, she had two episodes of high-rate atrial fibrillation (160 bpm) treated with metoprolol and amiodarone, with restoration of sinus rhythm. After hemodynamic stabilization, she was transferred to our ward for geriatric management.

**Table 1:** Main admission laboratory findings

Laboratory parameters	Laboratory findings
White blood cell count	6940/mm^3^
Red blood cell count	3,110,000/mm^3^
Hemoglobin	10.4 g/dl
Hematocrit	28.9%
Mean corpuscular volume	93 fl
Mean corpuscular hemoglobin	33.4 pg
Platelets count	223,000/mm^3^
Neutrophils	86%
Lymphocytes	8.9%
Monocytes	5%
Eosinophils	0%
Basophils	0.1%
Neutrophil count	5970/mmc
Lymphocyte count	620/mmc
Monocyte count	350/mmc
Eosinophil count	0/mmc
Basophil count	1/mmc
Erythrocyte sedimentation rate	27 mm/hour
Urea	53 mg/dl
Creatinine	1.14 mg/dl
Sodium	133 mEq/l
Potassium	4.1 mEq/l
N-terminal pro-B natriuretic peptide	3576 pg/ml
C-reactive protein	15.3 mg/dl
Pro-calcitonin	1.04 ng/ml
D-dimer	672 ng/ml
Aspartate aminotransferase	17 U/l
Alanine aminotransferase	35 U/l
High sensitivity troponin I	0.107 ng/ml
Creatine kinase	213 U/l

A real-time reverse transcriptase-polymerase chain reaction (rRT-PCR) assay for SARS-CoV-2 was not performed on admission, due to the lack of previous history of travels or close contact with a confirmed or probable case of COVID-19 in the previous 14 days, according to the epidemiological criteria disclosed by health authorities at that time (World Health Organization’s criteria of suspicion for SARS-CoV-2 infection). However, after her admission at our ward, the persistence of severe hypoxemia, fever (37.6 °C), cough, and high C-reactive protein (14.68 mg/dl) coupled with reduced pro-calcitonin (0.14 ng/ml) after appropriate antibiotic therapy, prompted us to collect nasopharyngeal and oropharyngeal swabs on 3 March 2020 even in the absence of epidemiological criteria. The rRT-PCR assay tested positive for SARS-CoV-2 infection. She was then isolated and managed by a dedicated health care team, according to the protocol of our hospital. After an initial period of clinical stability with high-flow oxygen, she required continuous positive airway pressure in order to maintain adequate oxygenation. On 25 March 2020 she died of cardiac complications.

## Discussion and conclusions

We described a case of a 95-year-old woman with several pre-existing comorbidities who was affected by COVID-19 pneumonia masked by aspiration pneumonia. If we were not in a SARS-CoV-2 pandemic, this would have been a classic case of aspiration pneumonia in a patient with vascular dementia and dysphagia. However, it turned out to be a case of COVID-19 pneumonia after the initial diagnosis. In our opinion, this case report highlights several important aspects of COVID-19.

On 2 March 2020, the date of admission to our ward, 1835 individuals had tested positive for SARS-CoV-2 at rRT-PCR assay in Italy, almost all concentrated in the Lombardy region, and only 35 cases had been tested positive in our smaller region (Marche), mainly in the northern part and not in our city [8]. Our patient was a bedridden older individual with very limited social contacts, who lived at home with her son and a caregiver who did not report any epidemiological link. Both individuals were asymptomatic. These findings probably indicate that SARS-CoV-2 had been circulating within the Italian population for some time previously, and they support the hypothesis that the virus was spreading undetected, probably through asymptomatic people. There is evidence that SARS-CoV-2 transmission can occur from asymptomatic or mildly symptomatic individuals [9, 10]. Some authors, using a model inference framework, estimated that 86% of all infections in China were undocumented prior to the travel restrictions of 23 January 2020, suggesting that undocumented infections might have been the source for 79% of documented cases [11]. This hypothesis could also be true for Italy. Findings from a population study in Vo’ Euganeo (Veneto region, Italy) showed that the majority of people infected with SARS-CoV-2 (50–75%) were asymptomatic, probably representing “a formidable source” of contagion [12].

Retrospective studies on Chinese patients hospitalized for COVID-19 showed that the disease has different features in older patients. In fact, they had higher disease severity compared to young and middle-aged patients, with higher Pneumonia Severity Index (PSI) score, higher proportion of multiple lobe involvement, higher C-reactive protein, and lower lymphocytes count [13]. Symptoms at onset of COVID-19 disease often include cough, dyspnea, and fever or measured temperature ≥ 38 °C. However, many older patients with pneumonia often exhibit atypical symptoms and signs compared to adults [14]. Older patients with pneumonia are often afebrile, with normal WBC count, while acute changes in functional and mental status are highly prevalent. Dyspnea could also be difficult to assess, given the limited physical activity of these individuals [14]. Given the possible atypical presentations, the diagnosis of pneumonia in older patients may be challenging. In a viral pandemic era, the clinical picture may be even more complicated. In our case, the diagnosis of aspiration pneumonia was supported by the presence of dysphagia, elevated C-reactive protein, and the findings at bronchoalveolar lavage. On the other hand, some other typical laboratory parameters of COVID-19 were also present, such as the increase in C-reactive protein-to-procalcitonin ratio, and absolute lymphopenia with normal WBC count [15]. However, these parameters in older patients may be difficult to interpret. For example, lymphopenia is very common in hospitalized older patients, representing a typical laboratory marker of frailty [16].

Radiographic findings of aspiration pneumonia include infiltrates in gravity-dependent lung segments (superior lower lobe or posterior upper lobe segments, if the patient is in a supine position during the event, or basal segments of the lower lobe, if the patient is upright during the event) [6]. On the other hand, GGOs and bilateral patchy shadowing, mainly in the lower lobes, are the most common patterns on chest CT in patients with COVID-19 [2, 17]. In fact, these CT abnormalities, not typically correlated to a diagnosis of aspiration pneumonia, were found in our case. The chest CT findings (multiple bilateral GGOs coupled with crazy-paving pattern and areas of consolidation) indicated that the COVID-19 had been present for at least approximately 5–7 days before the examination [18]. However, at that time, the experience of radiologists in interpreting and detecting COVID-19 pneumonia may have been limited by the absence of COVID-19 pneumonia spreading in our district. Furthermore, the interpretation of radiological findings can be complex in older patients [19]. In fact, a chest X-ray is often inconclusive in older patients with suspected acute lower respiratory infection [20]. At the same time, it could be difficult, even at a CT scan, to recognize the suspected pulmonary disease in the midst of the age-related changes of lung parenchyma and the several comorbidities that act as confounders [19]. For example, GGOs, a typical feature of COVID-19, have not been linked to age-related changes, but may be found in congestive heart failure, a very common condition in hospitalized older patients [21]. Our patient had high NT-proBNP levels on admission, indicative of decompensated heart failure. This finding is highly prevalent in older patients admitted for lung and other infections, but without an admission diagnosis of heart failure, and it predicts in-hospital mortality [21, 22]. The pro-calcitonin levels on admission (> 0.25 ng/ml) and the results of bronchoalveolar lavage probably indicated a bacterial infection [23], which further complicated the radiological picture of chest CT in our patient. The clinical picture may be further complicated by the low sensitivity of the rRT-PCR assay for SARS-CoV-2 tested by nasopharyngeal and oropharyngeal swabs [24].

During hospitalization, two paroxysms of high-rate atrial fibrillation occurred, which is a negative prognostic factor in COVID-19, together with both high NT-proBNP and troponin I levels [25]. This testifies how viral infection can worsen stable cardiovascular comorbidities, although direct myocardial damage due to SARS-CoV-2 has also been documented [26, 27]. Pre-existing conditions, especially cardiovascular and kidney diseases, are more prevalent in older patients with severe COVID-19 compared to patients with milder disease [2, 28]. These patients often die due to the worsening of these pre-existing conditions after the SARS-CoV-2 infection, resulting in multiple organ failure, just like other severe infections. The mortality rate of patients with acute kidney injury and COVID-19 illness is four times higher than in patients who do not have acute kidney injury [29]. Our patient had decompensated heart failure and died due to cardiac complications. There is still no consensus on the management of decompensated heart failure in older patients, particularly if affected by COVID-19. However, renin–angiotensin–aldosterone system blockers have been associated with lower in-hospital mortality in older patients admitted for medical conditions [30] and these drugs are also likely to be useful in the context of the COVID-19 pandemic [31]. Therefore, it is essential to evaluate carefully and treat comorbidities appropriately in patients with COVID-19, especially if older [32].

In conclusion, this case report highlights how the diagnosis of COVID-19 pneumonia could be challenging in comorbid older patients, given the possible atypical presentation and the overlapping of other acute and chronic conditions that may complicate the interpretation of clinical, radiological, and laboratory findings. Not least, this case report shows that probably there were undocumented cases of infections with a wider spread of the virus before we became aware of it.

## Data Availability

The dataset used during the current study is available from the corresponding author on reasonable request.

## Abbreviations

SARS-CoV-2: Severe acute respiratory syndrome coronavirus-2
COVID-19: Coronavirus disease 2019
BADL: Basic activities of daily living
bpm: Beats per minute
IADL: Instrumental activities of daily living
ISS: Italian National Institute of Health
CT: Computed tomography
GGOs: Ground glass opacities
WBC: White blood cell
NT-proBNP: N-terminal pro-B natriuretic peptide
rRT-PCR: Real-time reverse transcriptase-polymerase chain reaction
PSI: Pneumonia Severity Index
FiO_2_: Fraction of inspired oxygen
SaO_2_: Arterial oxygen saturation
